# Body Composition and Progression of Biopsy‐Proven Non‐Alcoholic Fatty Liver Disease in Patients With Obesity

**DOI:** 10.1002/jcsm.13605

**Published:** 2024-10-10

**Authors:** Qianyi Wan, Xingzhu Liu, Jinghao Xu, Rui Zhao, Shiqin Yang, Jianrong Feng, Zhan Cao, Jingru Li, Xiaopeng He, Haiou Chen, Jinbao Ye, Haiyang Chen, Yi Chen

**Affiliations:** ^1^ Department of General Surgery, Division of Gastrointestinal Surgery, West China Hospital Sichuan University Chengdu China; ^2^ West China School of Medicine Sichuan University Chengdu China; ^3^ Laboratory of Metabolism and Aging Research, National Clinical Research Center for Geriatrics and State key Laboratory of Respiratory Health and Multimorbidity, Frontiers Science Center for Disease‐Related Molecular Network, West China Hospital Sichuan University Chengdu China

**Keywords:** body composition, non‐alcoholic fatty liver disease, non‐alcoholic steatohepatitis, obesity

## Abstract

**Background:**

Obesity is a significant risk factor for the progression of non‐alcoholic fatty liver disease (NAFLD). However, a convenient and efficacious non‐invasive test for monitoring NAFLD progression in patients with obesity is currently lacking. This study aims to investigate the associations between CT‐based body composition and the progression of biopsy‐proven NAFLD in patients with obesity.

**Methods:**

Liver biopsy was conducted in patients with obesity, and the progression of NAFLD was evaluated by the NAFLD activity score (NAS). Body composition was assessed through abdominal computed tomography (CT) scans.

**Results:**

A total of 602 patients with an average age of 31.65 (±9.33) years old were included, comprising 217 male patients and 385 female patients. The wall skeletal muscle index (SMI), total SMI, and visceral fat index (VFI) were positively correlated with NAS in both male and female patients. Multivariate regression analysis demonstrated significant associations between high liver steatosis and wall SMI (HR: 1.60, 95% CI: 1.12 to 2.30), total SMI (HR: 1.50, 95% CI: 1.02 to 2.08), VSI (HR: 2.16, 95% CI: 1.48 to 3.14), visceral fat to muscle ratio (HR: 1.51, 95% CI: 1.05 to 2.18), and visceral to subcutaneous fat ratio (HR: 1.51, 95% CI: 1.07 to 2.12). Non‐alcoholic steatohepatitis (NASH) was significantly associated with wall SMI (HR: 1.52, 95% CI: 1.06 to 2.19) and VSI (HR: 1.50, 95% CI: 1.03 to 2.17). Liver fibrosis ≥ F2 was significantly associated with psoas muscle index (HR: 0.64, 95% CI: 0.44 to 0.93) and psoas skeletal muscle density (HR: 0.61, 95% CI: 0.41 to 0.89).

**Conclusions:**

Our study suggested that certain CT‐based body composition indicators, notably high VFI, were significantly associated with the progression of NAFLD in patients with obesity. Great attentions and timely managements should be given to these patients with body composition characteristics associated with the risk of NAFLD progression.

## 1 Introduction

Non‐alcoholic fatty liver disease (NAFLD), also known as metabolic dysfunction–associated steatotic liver disease (MASLD), stands as the prevailing chronic liver disorder globally [1, 2, 3]. It is estimated that the prevalence of NAFLD is approximately 25% worldwide [4]. Despite the classification of the majority of NAFLD as non‐alcoholic fatty liver (NAFL), a non‐progressive form of NAFLD. There are still about 20% NAFLD presenting as non‐alcoholic steatohepatitis (NASH) [4], which is a progressive and inflammatory liver disease with no approved pharmacological treatment [3, 5, 6]. In addition, a subset of NASH patients may further progress to significant liver fibrosis, cirrhosis, and even hepatocellular carcinoma, resulting in a grim prognosis [5, 6, 7]. Notably, obesity represents a significant risk factor for NAFLD and contributes to its progression [8]. A recent meta‐analysis of 151 studies revealed a worldwide prevalence of NAFLD as high as 75% in the obese population, with rates of NASH and significant liver fibrosis reaching 33.50% and 21.60%, respectively [9]. Therefore, early identification of patients with obesity at risk of NAFLD progression could facilitate timely management. Although liver biopsy is the reference standard for the evaluation of NAFLD progression, it is not suitable for wide‐scale population‐level screening [10]. Moreover, a convenient and efficacious non‐invasive test for monitoring NAFLD progression, especially in patients with obesity, is currently lacking.

Obesity is characterized by an excess accumulation of adipose tissue in the body [11]. The excessive build‐up of triglycerides in the liver is recognized as a pivotal occurrence in the initiation and progression of NAFLD [12]. Concurrently, obesity also promotes the accumulation of excess adipose tissue outside the liver, resulting in abnormal body composition [11]. Recent investigations have explored the potential correlations between body composition and NAFLD. For instance, a retrospective study by Xing et al. reported that a reduced appendicular skeletal muscle mass to visceral fat area ratio significantly correlated with a higher prevalence of NAFLD and liver fibrosis [13]. Concurrently, Yan et al. discovered a positive correlation between a higher fat‐to‐muscle ratio and an increased risk of NAFLD and liver fibrosis [14]. Furthermore, increased intermuscular adipose tissue and visceral adipose tissue were also identified as positively associated with NAFLD [15, 16]. However, most of these studies relied on ultrasonography, magnetic resonance imaging, fibrosis‐4 index, and NAFLD fibrosis score for diagnosing NAFLD and liver fibrosis, without a gold standard diagnosis based on liver biopsy. In addition, there is also a great deal of heterogeneity in the populations included in different studies. Therefore, there is a need for in‐depth investigation into the associations between body composition and progression of NAFLD in patients with obesity.

Considering that less is known about the associations between body composition and progression of NAFLD in patients with obesity, it is a need for in‐depth investigation in this issue. In this study, we aim to identify distinct body composition characteristics that exhibit notable associations with NAFLD progression. This identification could prove valuable in the timely identification and management of patients with obesity at risk of NAFLD progression.

## 2 Methods

### 2.1 Patient Selection

The patients with obesity receiving bariatric surgery between October 2019 and March 2024 in Division of Gastrointestinal Surgery, West China Hospital of Sichuan University were included in this study. The inclusion criteria were as follows: (1) adult patients; (2) liver biopsy was conducted during the bariatric surgery; (3) the abdominal computed tomography (CT) scan was performed in our hospital within 1 month before liver biopsy. In addition, the exclusion criteria were (1) alcohol consumption ≥30 g per day for men and ≥20 g per day for women [1]; (2) uncontrolled infectious liver disease or other chronic liver diseases; (3) a history of malignancy. We performed this study based on the Declaration of Helsinki, and it was approved by the ethics committee of West China Hospital.

### 2.2 Clinical Variables

The clinical data of the enrolled patients were acquired from medical records accessed via the Hospital Information System (HIS) of our hospital. The main clinical data in this study included gender, age, body mass index (BMI), cigarette smoking, and co‐morbidities (type 2 diabetes, hypertension, and dyslipidaemia). Additionally, we also gathered peripheral blood biochemical markers, comprising glucose, glycated haemoglobin (HbA1c), lipid‐related indicators, and liver function‐related indicators, derived from fasting venous blood assessments.

### 2.3 Liver Biopsy and Histology Assessment

All liver biopsies were conducted during the bariatric surgery, and the liver specimens were taken at the margin of left lobe of liver with scissors. All patients have signed informed consent forms before liver biopsies. Liver histology was examined according to the NAFLD activity score (NAS), which consists of steatosis (assessed on a scale of 0 to 3), lobular inflammation (assessed on a scale of 0 to 3), and hepatocellular ballooning (assessed on a scale of 0 to 2) [17]. NAFLD was defined as more than 5% hepatocyte steatosis [1, 7, 17]. In particular, hepatocyte steatosis ≤33% (corresponding to NAS of steatosis ≤1) [17] was recognized as low liver steatosis in this study. Liver fibrosis was classified into five categories (F0–F4), and fibrosis stage ≥F2 was referred to as clinically significant fibrosis [17, 18]. NASH was defined as NAS of ≥4, with fibrosis F1–F3 and at least a score of 1 in each of steatosis, ballooning, and lobular inflammation [17, 19].

### 2.4 Body Composition Analysis

A single CT image at the third lumbar vertebra (L3) level was derived from the preoperative abdominal CT scan, and the body composition analysis was conducted using a wildly applied cloud‐based AI platform *CoreSlicer* [20, 21, 22]. The cross‐sectional areas of psoas muscle, wall muscle, total muscle, subcutaneous fat, and visceral fat were identified and calculated according to the tissue‐specific Hounsfield unit (HU), and the HU threshold was set from −29 to 150 for skeletal muscle and −190 to −30 for adipose tissue, respectively [23, 24, 25]. The areas of each body composition were normalized by dividing the square of the height (cm^2^/m^2^), which subsequently created body composition parameters including psoas muscle index (PMI), wall skeletal muscle index (SMI), total SMI, subcutaneous fat index (SFI), and visceral fat index (VFI) [26]. The visceral fat to muscle ratio was calculated as the visceral fat area divided by total skeletal muscle area, and the visceral to subcutaneous fat ratio was the area of visceral fat divided by the subcutaneous fat area. Furthermore, skeletal muscle density (SMD), an indicator of the fat infiltration in skeletal muscle, was automatically generated as the mean radiation attenuation of the muscle region of interest (psoas SMD, wall SMD, and total SMD) [27].

### 2.5 Statistical Analysis

In this study, continuous variables were presented as mean plus standard deviation (SD) or median with range, and categorical data were shown as the number of cases. The *t*‐test or Mann–Whitney *U* test was conducted for the comparison of continuous data according to the normality, and the chi‐squared test or Fisher's exact test was conducted for the comparison for categorical data. Pearson's correlation analysis was performed to investigate the associations between NAS and body composition indicators. To further evaluate the associations between body composition indicators and liver steatosis, NASH, and liver fibrosis, the body composition indicators were transformed into binary data based on their median values in males and females, respectively. Subsequently, multivariate logistic regression analysis was applied with adjustments of gender, age, BMI, cigarette smoking, type 2 diabetes, hypertension, and dyslipidaemia, and the results were reported as hazard ratios (HR) and corresponding 95% confidence intervals (95% CI). All statistical analyses were conducted using the SPSS version 25.0 and GraphPad Prism version 8.0, and a two‐sided *P*‐value of <0.05 meant statistical significance in this study.

## 3 Results

### 3.1 Characteristics of Included Patients

**FIGURE 1:**
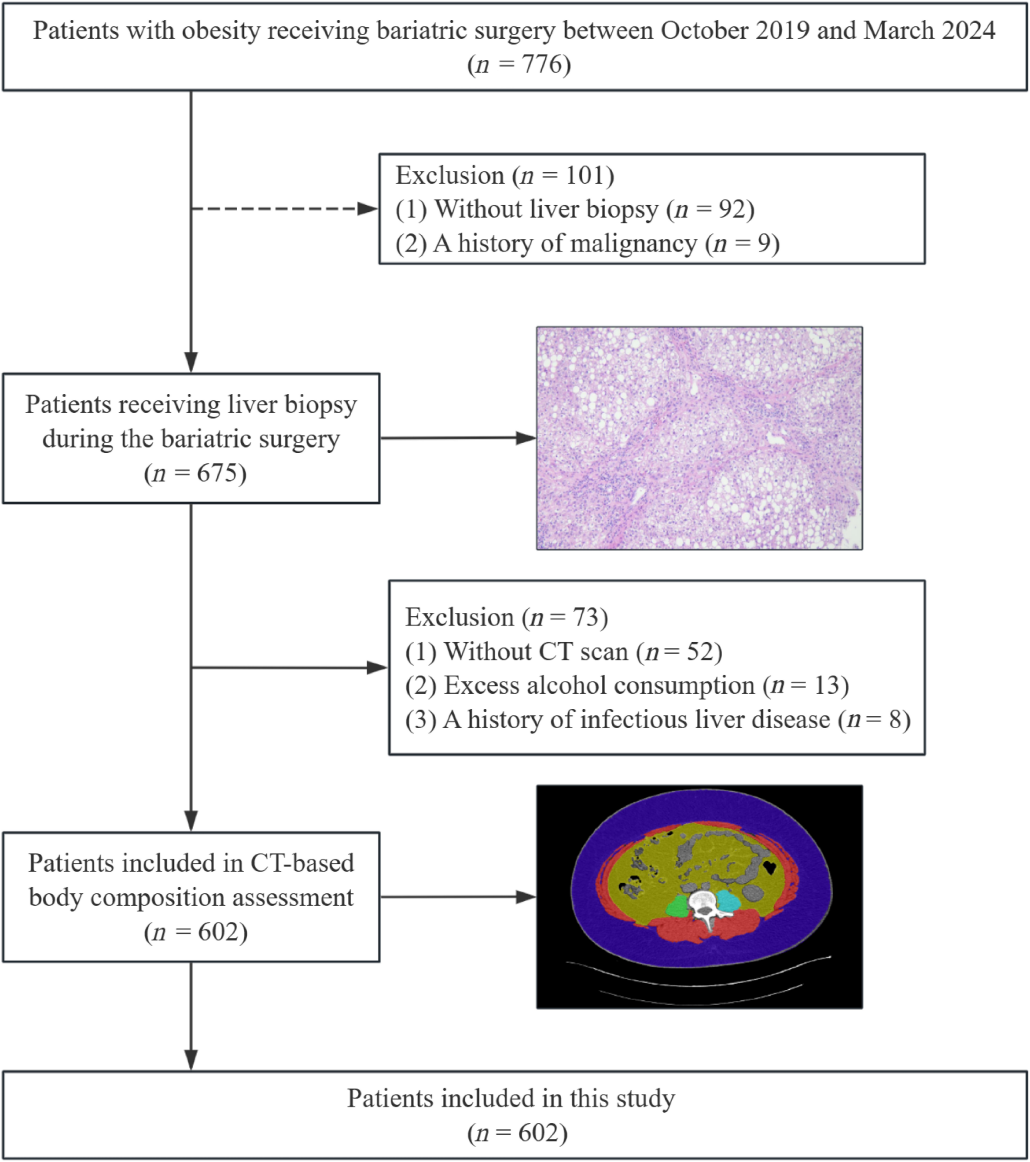
Flow diagram of patients.

In the preliminary assessment, 776 potentially eligible patients with obesity were initially identified, of which 174 patients did not meet the inclusion criteria and were consequently excluded from the study. Detailed patient screening process was shown in Figure 1. Eventually, a total of 602 patients with obesity were included, comprising 217 male patients and 385 female patients. The mean age of the included patients was 31.65 (±9.33), and the mean BMI was 36.76 (±5.38). Notably, 532 patients were diagnosed as NAFLD, representing 88.37% of the included populations.

We observed that male patients had a significantly higher prevalence of high liver steatosis, NASH, and liver fibrosis ≥ F2 (Table 1). Patients with high liver steatosis were notably younger than those with low liver steatosis, with no significant age difference in the subgroups of NASH and liver fibrosis. Besides, patients with high liver steatosis, NASH, or liver fibrosis ≥ F2 exhibited a significantly higher BMI and higher prevalence of type 2 diabetes and dyslipidaemia. Correspondingly, abnormal levels of circulatory glucose and lipid‐related biomarkers were observed in these patients (Table 1). Furthermore, the common liver function‐related indicators, including alanine aminotransferase, aspartate aminotransferase, and alkaline phosphatase, exhibited significant differences in each of the subgroup of liver steatosis, NASH, and liver fibrosis (Table 1). These results suggest that patients with progressive NAFLD, characterized by high liver steatosis, NASH, or liver fibrosis ≥ F2, are associated with more serious metabolic disorders.

**TABLE 1:** Clinical characteristics of included patients.

Characteristics	Liver steatosis			NASH			Liver fibrosis		
	Low (≤33%) (*n* = 340)	High (>33%) (*n* = 262)	*p*	None (*n* = 262)	Yes (*n* = 340)	*p*	<F2 (*n* = 426)	≥F2 (*n* = 176)	*p*
Male/female	99/241	118/144	<0.001	72/190	145/195	<0.001	132/294	85/91	<0.001
Age, mean ± SD (years)	32.50 ± 9.10	30.54 ± 9.51	0.01	31.92 ± 8.82	31.43 ± 9.70	0.52	31.40 ± 8.86	32.24 ± 10.37	0.32
BMI, mean ± SD	36.01 ± 5.36	37.73 ± 5.26	<0.001	35.56 ± 4.99	37.68 ± 5.50	<0.001	36.28 ± 5.14	37.93 ± 5.78	<0.001
Cigarette smoking (yes/no)	38/302	40/222	0.14	25/237	53/287	0.03	45/381	33/143	0.007
Type 2 diabetes (yes/no)	88/252	107/155	<0.001	52/210	143/197	<0.001	103/323	92/84	<0.001
Hypertension (yes/no)	46/294	31/231	0.54	22/240	55/285	0.005	45/381	32/144	0.01
Dyslipidaemia (yes/no)	129/211	131/131	0.003	96/166	164/176	0.004	165/261	95/81	<0.001
Glucose, mean ± SD (mmol/L)	6.21 ± 2.16	6.97 ± 2.77	<0.001	6.00 ± 1.77	6.96 ± 2.83	<0.001	6.25 ± 2.23	7.23 ± 2.86	<0.001
HbA1c, mean ± SD (%)	6.08 ± 1.28	6.53 ± 1.51	<0.001	5.96 ± 1.08	6.52 ± 1.56	<0.001	6.09 ± 1.26	6.72 ± 1.61	<0.001
ALT, mean ± SD (IU/L)	40.96 ± 34.27	71.38 ± 54.17	<0.001	40.82 ± 37.62	64.50 ± 50.03	<0.001	48.15 ± 39.12	68.84 ± 58.42	<0.001
AST, mean ± SD (IU/L)	27.18 ± 14.81	41.95 ± 28.95	<0.001	26.83 ± 15.17	38.83 ± 26.83	<0.001	30.38 ± 17.62	41.41 ± 31.91	<0.001
ALP, mean ± SD (IU/L)	76.27 ± 21.26	82.06 ± 28.06	0.004	74.83 ± 20.77	81.84 ± 26.81	<0.001	76.51 ± 20.66	84.32 ± 31.58	<0.001
TG, mean ± SD (nmol/L)	1.73 ± 1.33	2.33 ± 2.79	<0.001	1.74 ± 1.21	2.19 ± 2.59	0.01	1.93 ± 2.25	2.16 ± 1.73	0.22
CHO, mean ± SD (mmol/L)	4.67 ± 0.95	4.75 ± 1.04	0.34	4.75 ± 0.94	4.67 ± 1.03	0.33	4.73 ± 0.96	4.64 ± 1.06	0.32
HDL, mean ± SD (mmol/L)	1.16 ± 0.26	1.06 ± 0.21	<0.001	1.17 ± 0.25	1.07 ± 0.23	<0.001	1.14 ± 0.25	1.05 ± 0.22	<0.001
LDL, mean ± SD (mmol/L)	2.95 ± 0.86	2.91 ± 0.87	0.66	3.03 ± 0.86	2.86 ± 0.86	0.02	2.96 ± 0.85	2.85 ± 0.89	0.15
TBA, mean ± SD (μmol/L)	2.91 ± 4.19	3.29 ± 3.74	0.26	2.64 ± 2.23	3.41 ± 4.93	0.02	2.71 ± 2.53	3.94 ± 6.20	<0.001

Abbreviations: ALP, alkaline phosphatase; ALT, alanine aminotransferase; AST, aspartate aminotransferase; BMI, body mass index; CHO, cholesterol; HbA1c, glycated haemoglobin; HDL, high‐density lipoprotein; LDL, low‐density lipoprotein; NASH, non‐alcoholic steatohepatitis; SD, standard deviation; TBA, total bile acid; TG, triglyceride.

### 3.2 Correlations Between Body Composition and NAS

**FIGURE 2:**
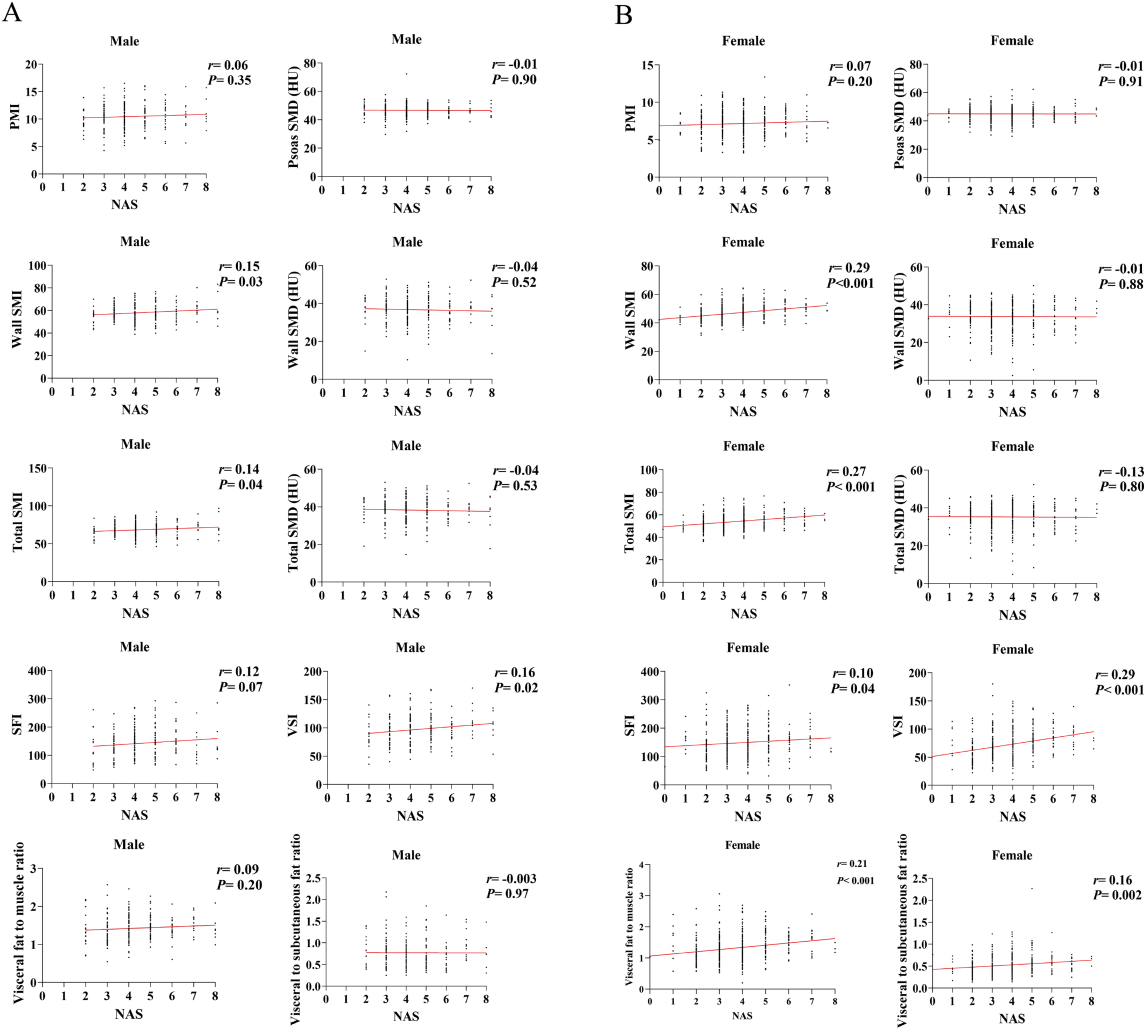
Correlations between body composition indicators and non‐alcoholic fatty liver disease activity score in (A) male patients; (B) female patients.

The body composition indicators we investigated in this study included PMI, psoas SMD, wall SMI, wall SMD, total SMI, total SMD, SFI, VSI, visceral fat to muscle ratio, and visceral to subcutaneous fat ratio. Notably, significant differences in all body composition indicators, except for SFI, were observed between male and female patients (Table 2). Consequently, the correlations between body composition and NAS were investigated separately for male and female patients. We found that several body composition indicators, including wall SMI, total SMI, and VSI, exhibited significant positive correlations with NAS in both male (Figure 2A) and female (Figure 2B) patients. Besides, the SFI, visceral fat to muscle ratio, and visceral to subcutaneous fat ratio were positively correlated with NAS in females (Figure 2B) but not in males (Figure 2A), while no significant correlations were observed between the remaining body composition indicators and NAS (Figure 2A, B). These results indicated that certain body composition indicators were significantly correlated with the progression of NAFLD.

**TABLE 2:** Comparison of body composition between male and female patients.

Characteristics	Male (*n* = 217)	Female (*n* = 385)	*p*
Age, mean ± SD (years)	31.31 ± 9.62	31.84 ± 9.16	0.51
BMI, mean ± SD	38.73 ± 5.81	35.65 ± 4.79	<0.001
PMI, mean ± SD	10.46 ± 2.32	7.15 ± 1.64	<0.001
Psoas SMD (HU), mean ± SD	46.69 ± 4.74	44.97 ± 4.63	<0.001
Wall SMI, mean ± SD	58.06 ± 7.81	46.99 ± 5.79	<0.001
Wall SMD (HU), mean ± SD	36.79 ± 6.91	33.79 ± 6.95	<0.001
Total SMI, mean ± SD	68.53 ± 9.20	54.13 ± 6.73	<0.001
Total SMD (HU), mean ± SD	38.30 ± 6.35	35.24 ± 6.56	<0.001
SFI, mean ± SD	142.65 ± 51.87	148.97 ± 51.52	0.15
VSI, mean ± SD	97.16 ± 25.26	71.64 ± 26.16	<0.001
Visceral fat to muscle ratio, mean ± SD	1.43 ± 0.35	1.32 ± 0.45	0.003
Visceral to subcutaneous fat ratio, mean ± SD	0.77 ± 0.35	0.52 ± 0.23	<0.001

Abbreviations: BMI, body mass index; HU, Hounsfield unit; PMI, psoas muscle index; SD, standard deviation; SFI, subcutaneous fat index; SMD, skeletal muscle density; SMI, skeletal muscle index; VSI, visceral fat index.

### 3.3 Associations Between Body Composition and High Liver Steatosis, NASH, and Significant Liver Fibrosis

**FIGURE 3:**
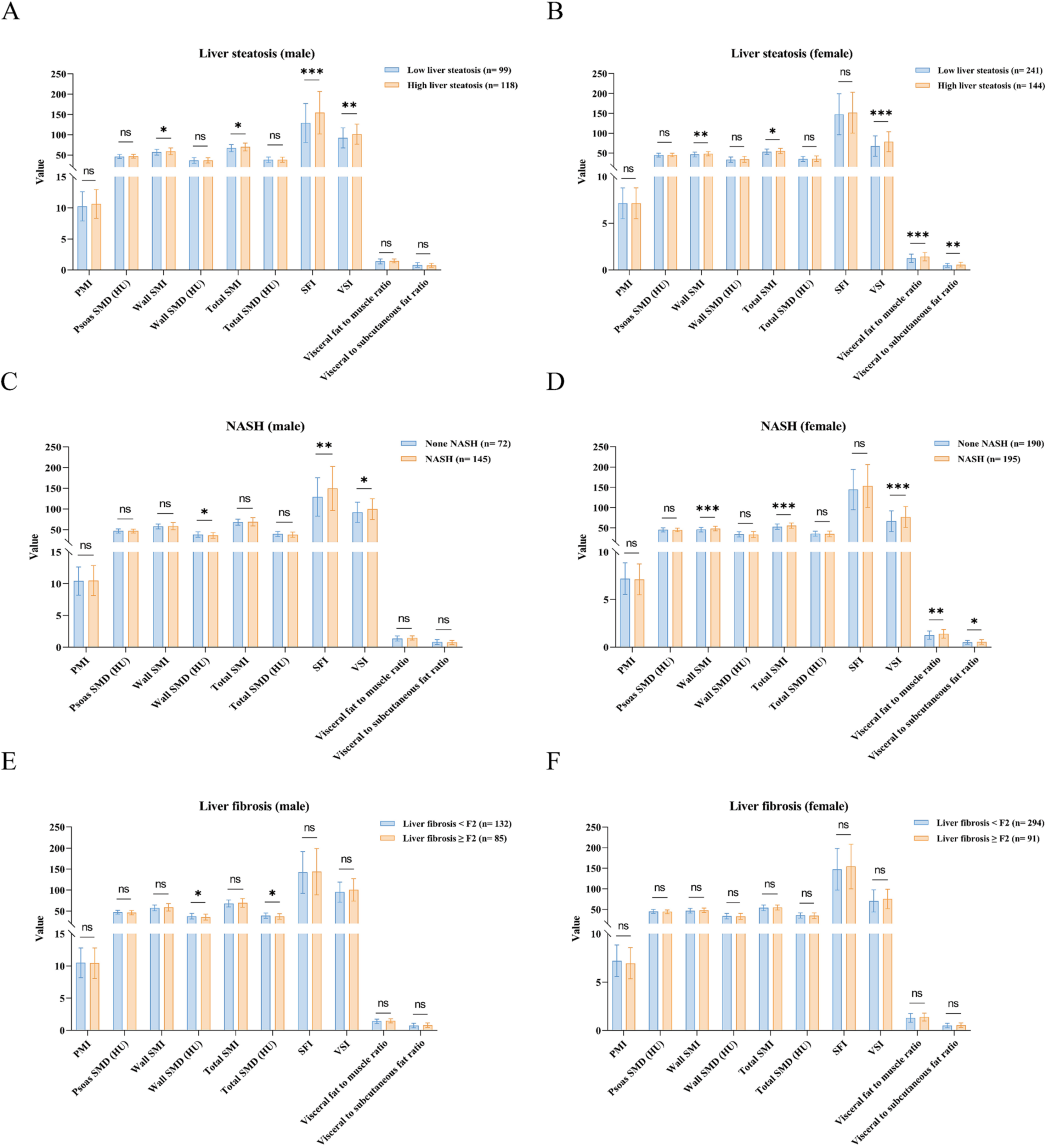
Comparison of body composition indicators in subgroup of: (A) male liver steatosis; (B) female liver steatosis; (C) male non‐alcoholic steatohepatitis; (D) female non‐alcoholic steatohepatitis; (E) male liver fibrosis; (F) female liver fibrosis.

Hepatocyte steatosis is the most characteristic of NAFLD. In male patients, those with high liver steatosis had a significantly higher value of wall SMI, total SMI, SFI, and VSI compared to those with low liver steatosis (Figure 3A). Furthermore, female patients with high liver steatosis exhibited a significantly higher value of wall SMI, total SMI, VSI, visceral fat to muscle ratio, and visceral to subcutaneous fat ratio (Figure 3B). In multivariate analysis, after adjustments of gender, age, BMI, cigarette smoking, type 2 diabetes, hypertension, and dyslipidaemia, we found that high liver steatosis was significantly associated with wall SMI (HR: 1.60, 95% CI: 1.12 to 2.30, *p* = 0.01), total SMI (HR: 1.50, 95% CI: 1.02 to 2.08, *p* = 0.04), VSI (HR: 2.16, 95% CI: 1.48 to 3.14, *p* < 0.001), visceral fat to muscle ratio (HR: 1.51, 95% CI: 1.05 to 2.18, *p* = 0.03), and visceral to subcutaneous fat ratio (HR: 1.51, 95% CI: 1.07 to 2.12, *p* = 0.02) (Table 3).

**TABLE 3:** Multivariate regression analysis of body composition and progression of non‐alcoholic fatty liver disease.

Characteristics	High liver steatosis			NASH			Liver fibrosis ≥ F2		
	HR^a^	95% CI	*p*	HR^a^	95% CI	*p*	HR^a^	95% CI	*p*
PMI (<median as ref)	1.02	0.72 to 1.43	0.93	0.84	0.60 to 1.19	0.33	0.64	0.44 to 0.93	0.02
Psoas SMD (HU) (<median as ref)	0.92	0.65 to 1.30	0.63	0.76	0.54 to 1.08	0.12	0.61	0.41 to 0.89	0.01
Wall SMI (<median as ref)	1.60	1.12 to 2.30	0.01	1.52	1.06 to 2.19	0.02	1.28	0.86 to 1.91	0.22
Wall SMD (HU) (<median as ref)	1.10	0.78 to 1.56	0.58	0.88	0.62 to 1.25	0.47	0.85	0.58 to 1.24	0.40
Total SMI (<median as ref)	1.50	1.02 to 2.08	0.04	1.41	0.98 to 2.01	0.06	1.16	0.78 to 1.71	0.47
Total SMD (HU) (<median as ref)	1.20	0.85 to 1.70	0.29	0.90	0.64 to 1.28	0.57	0.81	0.55 to 1.18	0.27
SFI (<median as ref)	1.30	0.86 to 1.97	0.22	1.10	0.72 to 1.68	0.67	0.66	0.42 to 1.06	0.08
VSI (<median as ref)	2.16	1.48 to 3.14	<0.001	1.50	1.03 to 2.17	0.03	1.13	0.75 to 1.70	0.55
Visceral fat to muscle ratio (<median as ref)	1.51	1.05 to 2.18	0.03	1.33	0.92 to 1.92	0.13	0.97	0.65 to 1.45	0.89
Visceral to subcutaneous fat ratio (<median as ref)	1.51	1.07 to 2.12	0.02	1.35	0.95 to 1.91	0.10	0.89	0.61 to 1.30	0.54

Abbreviations: 95% CI, 95% confidence intervals; HR, hazard ratios; HU, Hounsfield unit; NASH, non‐alcoholic steatohepatitis; PMI, psoas muscle index; SFI, subcutaneous fat index; SMD, skeletal muscle density; SMI, skeletal muscle index; VSI, visceral fat index.

^a^ Adjustments were made for gender, age, BMI, cigarette smoking, type 2 diabetes, hypertension, and dyslipidaemia.

For NASH, we observed that male patients with NASH were significantly associated with a higher value of SFI, VSI, and a lower value of wall SMD compared to those without NASH (Figure 3C). While female patients with NASH exhibited a significantly higher value of wall SMI, total SMI, VSI, visceral fat to muscle ratio, and visceral to subcutaneous fat ratio (Figure 3D). Multivariate analysis revealed that NASH was significantly associated with wall SMI (HR: 1.52, 95% CI: 1.06 to 2.19, *p* = 0.02) and VSI (HR: 1.50, 95% CI: 1.03 to 2.17, *p* = 0.03) (Table 3).

Liver fibrosis is one of the direct factors leading to poor prognosis in patients with NAFLD [1, 7]. In male patients, those with liver fibrosis ≥ F2 had a significantly lower value of wall SMD and total SMD compared to those with liver fibrosis < F2 (Figure 3E). While in female patients, there was no significant difference in each body composition indicator between patients with and without significant liver fibrosis (Figure 3F). Notably, in multivariate analysis, liver fibrosis ≥ F2 was only significantly associated with PMI (HR: 0.64, 95% CI: 0.44 to 0.93, *p* = 0.02) and psoas SMD (HR: 0.61, 95% CI: 0.41 to 0.89, *p* = 0.01) (Table 3).

## 4 Discussion

The present study of 602 patients with obesity identified significant associations between body composition and progression of biopsy‐proven NAFLD. A notable result of this study was that VSI had significantly positive correlations with NAS in both male and female patients. In addition, we found that patients with high liver steatosis or NASH had a significantly higher value of VSI regardless of the gender. Meanwhile, multivariate regression analysis also identified that a high VSI but not SFI was significantly associated with increased incidences of high liver steatosis and NASH. Visceral fat represents a crucial component of the abdominal adipose depot. Noteworthy is the finding that an excess of visceral fat, rather than subcutaneous fat, exhibited a positive correlation with ectopic fat deposition in various organs such as the pancreas, liver, renal sinus, and skeletal muscle [28]. A study involving 40 subjects who underwent liver biopsy demonstrated a higher mass of visceral fat in those with NAFLD [29]. In addition, another study of 2482 participants indicated that those with CT‐determined liver steatosis had higher volume of visceral fat [30]. Furthermore, concomitant alterations in liver fat content and visceral fat were noted following interventions for NAFLD. For instance, a clinical trial of 41 children with obesity reported that 9‐day isocaloric fructose restriction decreased both liver fat and visceral fat [31]. Another clinical trial found that ipragliflozin exerted significant beneficial effects on NAFLD, as well as reducing the visceral fat [32]. These studies revealed significant associations between visceral fat and the incidence of NAFLD, and our study further identified that excessive visceral fat was highly associated with the progression of NAFLD in patients with obesity.

Another notable finding of this study was that abdominal wall SMI had significantly positive correlations with NAS regardless of the gender, and a high wall SMI was significantly associated with increased incidences of high liver steatosis and NASH as indicated in multivariate regression analysis. Our findings align with a prior investigation involving 138 patients with obesity, which reported that the SMI assessed by bioelectrical impedance analysis (BIA) was significantly higher in those with biopsy‐proved NASH [33]. Furthermore, the multivariate regression analysis of our study found that high psoas SMD were significantly associated with a decreased incidence of liver fibrosis ≥ F2. Similarly, a study of 104 patients with obesity also identified that psoas muscle fat infiltration was associated with a 2.9‐fold increased risk for significant liver fibrosis [34]. These results indicated that skeletal muscle‐related body composition indicators were significantly associated with the progression of NAFLD. Nevertheless, controversies exist regarding the relationship between skeletal muscle mass and NAFLD in contemporary researches. For instance, a few studies reported that sarcopenia and low SMI were significantly associated with increased risks of NAFLD and NASH [35, 36, 37]. Considering the significant differences in existing research on the diagnosis of fatty liver, methods of skeletal muscle measurement, and characteristics of study populations, it is necessary to carefully evaluate the target population and reference value of these results.

Obesity is acknowledged as the major risk factor for promoting NAFLD progression, such as increasing the risk of NASH and liver fibrosis [9]. Notably, there is no approved pharmacological treatment for the NASH, with effective treatments for advanced liver fibrosis limited to liver transplantation [6, 38]. Hence, early identification of individuals at high risk of NAFLD progression and timely managements are of vital importance for preventing NAFLD progression, particularly for those with obesity. Several clinical guidelines advocate for dietary restrictions, increased physical activity, and pharmacological management of diabetes in the treatment of NAFLD [7, 39, 40] [S41]. Additionally, a few ongoing clinical trials are investigating the effects of metabolic modulators and anti‐inflammatory agents, including semaglutide and tirzepatide, on the improvement of NASH.^s42, s43^ Notably, bariatric surgery, the most effective strategy for the treatment of obesity and type 2 diabetes mellitus,^s44^ also shows great effect on preventing NAFLD progression. A recent multicentre, randomized trial identifies that bariatric surgery is more effective than lifestyle interventions and optimized medical therapy in the treatment of NASH and preventing the progression of liver fibrosis.^s45^ Consequently, the clinical value of our study lies in discerning the body composition risks associated with NAFLD progression in patients with obesity, and we suggest that those at risk of NAFLD progression might actively undergo bariatric surgery.

The main strength of our study was that we included a relatively large sample of patients with liver biopsy. Due to the relatively high risk of bleeding and lack of appropriate indications, traditional percutaneous liver biopsies are difficult to apply in early‐stage asymptomatic patients with NAFLD.^s46^ Conversely, in our study, liver biopsies were routinely performed for all patients with obesity undergoing bariatric surgery. Therefore, we are able to encompass a relatively complete disease spectrum of NAFLD, including early‐stage NAFLD and even individuals without NAFLD. Furthermore, liver specimens were obtained with scissors during bariatric surgery, yielding well‐structured chunks of liver tissues and ensuring a safer procedure compared to percutaneous liver biopsies [S47]. Additionally, abdominal CT scans of all participants were uniformly conducted in our hospital, and body composition indicators were analysed consistently to minimize bias. Nevertheless, limitations exist in this study. Due to constraints of the abdominal CT scan, body composition indicators were assessed based on the third lumbar vertebra level, neglecting the analysis of skeletal muscle and fat content in the limbs. Even so, the CT scan of the third lumbar vertebra level has been widely recognized as a standard method for analysing body compositions, which significantly correlates with whole‐body skeletal muscle and adipose tissue [S48, S49]. Moreover, numerous high‐quality clinical studies have identified the reliability of this method in assessing skeletal muscle, subcutaneous fat, and visceral fat [23, 24, 25, 26]. Furthermore, this is a single‐centre study, with a relatively concentrated geographical distribution of participants. Given that the type of obesity can be influenced by geographic and ethnic factors [S50, S51], future studies involving diverse populations of obesity are necessary to confirm our findings.

In summary, our study suggested that certain CT‐based body composition indicators, notably high VFI, were significantly associated with the progression of NAFLD in patients with obesity. Therefore, great attentions and timely managements should be given to these patients with body composition characteristics associated with the risk of NAFLD progression.

## Conflicts of Interest

The authors declare no conflicts of interest.

## Supporting information


**Data S1.** Supporting Information

## Data Availability

All data generated or analysed during this study are included in this published article.
