# Supplementary material for: Body Composition and Progression of Biopsy‐Proven Non‐Alcoholic Fatty Liver Disease in Patients With Obesity
Source: J Cachexia Sarcopenia Muscle. 2024 Oct 10;15(6):2608–17. doi: 10.1002/jcsm.13605 (PMC11634503; doi:10.1002/jcsm.13605)
Supplement: Supplementary file 1 — Data S1. Supporting Information [file JCSM-15-2608-s001.docx]

Supplementary references:

S41. Chalasani N, Younossi Z, Lavine JE, et al. The diagnosis and management of nonalcoholic fatty liver disease: Practice guidance from the American Association for the Study of Liver Diseases. Hepatology 2018;67:328-357.

S42. Newsome PN, Buchholtz K, Cusi K, et al. A Placebo-Controlled Trial of Subcutaneous Semaglutide in Nonalcoholic Steatohepatitis. N Engl J Med 2021;384:1113-1124.

S43. Hartman ML, Sanyal AJ, Loomba R, et al. Effects of Novel Dual GIP and GLP-1 Receptor Agonist Tirzepatide on Biomarkers of Nonalcoholic Steatohepatitis in Patients With Type 2 Diabetes. Diabetes Care 2020;43:1352-1355.

S44. Sandoval DA, Patti ME. Glucose metabolism after bariatric surgery: implications for T2DM remission and hypoglycaemia. Nat Rev Endocrinol 2023;19:164-176.

S45. Verrastro O, Panunzi S, Castagneto-Gissey L, et al. Bariatric-metabolic surgery versus lifestyle intervention plus best medical care in non-alcoholic steatohepatitis (BRAVES): a multicentre, open-label, randomised trial. Lancet 2023;401:1786-1797.

S46. Sanyal AJ, Shankar SS, Yates KP, et al. Diagnostic performance of circulating biomarkers for non-alcoholic steatohepatitis. Nat Med 2023;29:2656-2664.

S47. Barbois S, Arvieux C, Leroy V, et al. Benefit-risk of intraoperative liver biopsy during bariatric surgery: review and perspectives. Surg Obes Relat Dis 2017;13:1780-1786.

S48. Rinninella E, Cintoni M, Raoul P, et al. Muscle mass, assessed at diagnosis by L3-CT scan as a prognostic marker of clinical outcomes in patients with gastric cancer: A systematic review and meta-analysis. Clin Nutr 2020;39:2045-2054.

S49. Catanese S, Aringhieri G, Vivaldi C, et al. Role of Baseline Computed-Tomography-Evaluated Body Composition in Predicting Outcome and Toxicity from First-Line Therapy in Advanced Gastric Cancer Patients. J Clin Med 2021;10.

S50. Tu WJ, Zhao Z, Yan F, et al. Geographic and ethnicity variation in the prevalence of middle-aged and elderly adult obesity in China in 2020. Diabetes Obes Metab 2024;26:1897-1907.

S51. Liu J, Lavie CJ, Park YM, et al. Geographic variation and trends in prevalence of obesity among US adolescents, 2016-2021. Public Health 2023;223:128-130.
